# Rare disease emerging as a global public health priority

**DOI:** 10.3389/fpubh.2022.1028545

**Published:** 2022-10-18

**Authors:** Claudia Ching Yan Chung, Hong Kong Genome Project, Annie Tsz Wai Chu, Brian Hon Yin Chung

**Affiliations:** ^1^Hong Kong Genome Institute, Hong Kong, Hong Kong SAR, China; ^2^Department of Paediatrics and Adolescent Medicine, School of Clinical Medicine, Li Ka Shing Faculty of Medicine, The University of Hong Kong, Hong Kong, Hong Kong SAR, China

**Keywords:** rare disease, genomic equity, diversity, public health priority, inclusiveness, Hong Kong Genome Project

## Abstract

The genomics revolution over the past three decades has led to great strides in rare disease (RD) research, which presents a major shift in global policy landscape. While RDs are individually rare, there are common challenges and unmet medical and social needs experienced by the RD population globally. The various disabilities arising from RDs as well as diagnostic and treatment uncertainty were demonstrated to have detrimental influence on the health, psychosocial, and economic aspects of RD families. Despite the collective large number of patients and families affected by RDs internationally, the general lack of public awareness and expertise constraints have neglected and marginalized the RD population in health systems and in health- and social-care policies. The current Coronavirus Disease of 2019 (COVID-19) pandemic has exposed the long-standing and fundamental challenges of the RD population, and has reminded us of the critical need of addressing the systemic inequalities and widespread disparities across populations and jurisdictions. Owing to the commonality in goals between RD movements and universal health coverage targets, the United Nations (UN) has highlighted the importance of recognizing RDs in policies, and has recently adopted the UN Resolution to promote greater integration of RDs in the UN agenda, advancing UN's commitment in achieving the 2030 Sustainable Development Goals of “leav[ing] no one behind.” Governments have also started to launch Genome Projects in their respective jurisdictions, aiming to integrate genomic medicine into mainstream healthcare. In this paper, we review the challenges experienced by the RD population, the establishment and adoption of RD policies, and the state of evidence in addressing these challenges from a global perspective. The Hong Kong Genome Project was illustrated as a case study to highlight the role of Genome Projects in enhancing clinical application of genomic medicine for personalized medicine and in improving equity of access and return in global genomics. Through reviewing what has been achieved to date, this paper will provide future directions as RD emerges as a global public health priority, in hopes of moving a step toward a more equitable and inclusive community for the RD population in times of pandemics and beyond.

## Introduction

**Figure 1:**
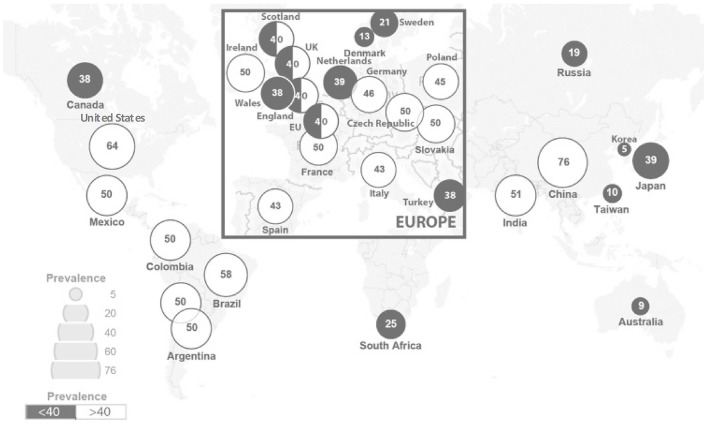
RD prevalence per 100,000 across jurisdictions [adapted from Richter et al. (9)].

Rare diseases (RDs) are an emerging public health priority. RD refers to a disease that affects a small number of people in a population (1). There are 6,000–8,000 unique RDs identified, with approximately 80% being genetic in origin, and 50–75% being pediatric onset (1–3). They are often chronic, progressive, and debilitating, and can lead to significant morbidity and mortality (4). With RDs' nature being heterogeneous, complex, and individually rare, they are difficult to be diagnosed, and are challenging to be assessed in aggregate. Currently there is no universal definition for RDs, with differing prevalence among different parts of the world. The European Union Regulation on orphan medicinal products defined RDs as conditions affecting < 50 per 100,000 individuals in the European population (5), whereas the American Orphan Drug Act defined RDs as conditions affecting < 200,000 individuals in the United States (6, 7). Other definitions have been proposed by different jurisdictions, ranging from five per 100,000 to 76 per 100,000 individuals, with the global average being 40 per 100,000 individuals (Figure 1) (8–10).

Although individually rare, the collective number of people affected by RDs was equivalent to the population of the world's third largest country (11). A recent global RD prevalence based on 3,585 RDs was estimated to be 3.5–5.9% of the world's population, which corresponds to 263 to 446 million people worldwide (10). When the impact of RDs extends to family members and carers of the RD patient, it was expected that RDs affect approximately 1.05–1.4 billion people globally (12).

Long diagnostic odyssey, lifelong disabilities, lack of compensatory support, and few but costly effective treatments are some of the unmet needs that plagues the lives of RD patients (13, 14). The various disabilities arising from the disease as well as diagnostic and treatment uncertainty have been demonstrated to have detrimental influence on the health, psychosocial, and economic aspects of the lives of the RD families (15, 16). In 2019, Rare Diseases International released a position paper emphasizing the need for universal health coverage (UHC) policies to account for RDs, owing to the commonality in goals between RD movements and the UHC targets (17). The United Nations (UN) political declaration on UHC has recognized the RD population as a marginalized group that should be considered during healthcare planning, claiming that UHC “shall never be fully attained nor realized if persons living with RDs are left behind and their needs left unmet” (17). Despite the significant challenges faced, under allocation of resources and inadequate healthcare planning for the RD population remains prevalent (13).

## Challenges experienced by the RD population

While RDs are individually rare, there are common features across the range of RDs and common challenges experienced by the RD population. Unmet medical and social needs of RD patients, families, and carers exist globally. Approximately half of the individuals with suspected RDs are undiagnosed, while RD patients who have received a diagnosis encounter fundamental myriad challenges due to delays or incorrect diagnoses, treatment, care, and social acceptance (18).

From the individual's perspective, long diagnostic odyssey often plagues the lives of RD patients. In Europe, 25% of the RD patients had to wait between 5 and 30 years from disease onset to receiving a genetic diagnosis for their condition, and 40% had initially received multiple misdiagnoses, leading to ineffective and unnecessary medical management (19). In another survey of RD families from the United Kingdom and United States, patients typically visit eight physicians and receive two to three misdiagnoses prior to receiving a correct genetic diagnosis, which spanned over a period of 5.6–7.6 years (2, 20). Not only do the individuals endure years of diagnostic odyssey, but it is also expensive for the health systems to undergo a succession of unnecessary medical follow-ups and conventional diagnostic approaches.

Undeniably, a genetic diagnosis offers the potential for personalized medicine, yet opens another door of challenges in treatment availability, accessibility, and affordability. With RDs being heterogeneous and individually rare, interventions and therapies, including orphan drugs, are seldom available due to the lack of market incentives and small market opportunity for the biopharmaceutical industry (21, 22). Currently, < 3% of diagnosed RDs have a suitable drug treatment (21, 23); it was estimated that fewer than one-tenth of RD patients have received disease-specific treatment globally (24). Where a treatment has been approved for a RD, cost of the drug is generally extremely costly, with RD drugs reported to be as high as 13.8 times more than conventional drugs (21, 23). This can be financially overwhelming for many, especially when RD drugs usually require out-of-pocket (OOP) cost-sharing by the patient. Consequently, RD patients may need to bear the catastrophically high OOP expenditure on health services and resources, posing a higher risk of financial hardship. For patients who are not able to afford the extremely costly therapies, they will continue to be managed with conventional approaches, adding to the never-ending socio-economic costs of RDs. Accessibility also remains to be a problem, with access and reimbursement recommendations on the same intervention varying vastly across jurisdictions.

From a wider socio-economic perspective, both patients and carers have highlighted the challenges in maintaining employment and education due to frequent medical follow-ups and the unprecedented and uncertainty nature of their condition (25–28). In the United Kingdom, 66% of the RD patients and carers indicated that their ability to hold paid employment was affected, with many of them being forced to retire early or reduce working hours due to the condition or the related caring responsibilities (25). Importantly, a significant number of RD patients and carers were forced to reduce working days or quit their job completely by their employer because they were considered as “unreliable.” These ultimately manifest as significant opportunities and productivity loss, and can be a burden for the RD patients, families, and the society as a whole. Due to social discrimination and stigmatization, low social awareness, and lack of knowledge and understanding from the general public, both RD patients and carers often feel isolated and excluded from the society (29). As such, the RD population experience extraordinary healthcare, psychosocial, and economic burden, contributing to the decreased wellbeing and quality of life.

Despite the collective large number of patients and families affected by RDs internationally, the general lack of public awareness and expertise constraints have neglected and marginalized the RD population in healthcare systems and in health- and social-care policies.

### Challenges in funding treatments and therapies for RDs

In the era of resource and budget constraints, health economic evidence plays a critical role in guiding decision makers to prioritize and allocate resources efficiently and effectively. Although cost-effectiveness and cost-utility analyses are often considered to be more useful in informing health and social care decisions, as they take account into both costs and outcomes simultaneously as compared to other option alternatives, such types of analyses are relatively difficult to be conducted within the RD population. This is due to the limited intervention alternatives that are available in the market, the small number of patients that can be recruited into clinical studies, and the conflicting ethical considerations for funding RD treatment (21, 22, 30, 31). Orphan drugs and RD interventions are often considered to be cost-ineffective against standard cost-effectiveness thresholds, such as the £20k – £30k (US$26k–$39k) per quality-adjusted life year (QALY) threshold proposed by National Institute for Health and Care Excellence (NICE), due to treatment's epidemiological and economic specifics (31, 32). Future health technology assessments concerning epidemiological, clinical, and economic evidence are warranted for assessing and appraising RD treatment and medications at a territory-wide or national level (31).

Genomic medicine challenges conventional health economic evaluation paradigms, which fails to capture the multi-dimensional outcomes that genomic medicine generates. Some health economists and ethicists have argued for an adjusted threshold for the RD population, such as the £78.3k (US$102.4k) per QALY threshold at the RD mid-point population and £937.1k (US$1,225.9k) per QALY for ultra-rare orphan drugs, based on the principles of equity and “veil of ignorance” (22, 33–35). Nevertheless, the adjusted threshold does not fully encompass the challenges associated with rarity. While the QALY can be a useful measure to evaluate health-related quality of life and survival, its simplicity in methodological calculation does not capture multi-dimensional patient benefits. The QALY is only one of the many elements of value in the “value flower” proposed by Lakdawalla et al., which could all contribute to how a healthcare intervention is valued (36). Elements such as the severity of disease, insurance value, real option value, and equity, are particularly relevant and important for RD therapies and should also be considered (37). Others have proposed that efficiency assessments such as cost per QALY should not be employed when the alternative choice is between an only treatment and no treatment (35). On the other hand, the multi-criteria decision analysis approach is proposed to provide more transparent and inclusive evidence in identifying and combining the relative importance of different criteria and stakeholder perspectives in a single health technology assessment for RD therapies, with the aim of balancing evidence among different stakeholders. More recently, the new NICE methods and processes for technology appraisals have been adopted in February 2022, with some of the changes made of particular relevance to determining the value of RD therapies. These include consideration of disease severity, different types of evidence including qualitative and expert elicitation, flexibility to accept uncertainty in specific situations, and commercial and managed access. It is recommended that NICE appraisals should consider the degree of need and desirability to promote innovation in addition to the clinical effectiveness and value for money. Health system's obligations for equality and human rights must also be considered. Flexibilities should be adopted rather than strictly following the cost-effectiveness threshold. These provide an innovative and sustainable framework to assess and appraise RD interventions. In the future, decision makers and health authorities should take account into the spill over effect, the broader social value of RD treatment and intervention, and their potential and innovativeness for other non-rare cases (31).

## RDs under the COVID-19 pandemic

The current Coronavirus Disease of 2019 (COVID-19) pandemic remains to be an unprecedented global health challenge due to its persistent spread and unpredictable clinical course. As of August 23, 2022, over 595.1 million cases were confirmed and over 6.4 million deaths were reported across 222 jurisdictions since the outbreak of COVID-19 in December 2019 in Wuhan China (38). The pandemic has reminded us of the critical need of addressing the systemic inequalities in the determinants of health and illnesses, including genomic, social, and environmental factors, which has resulted in widespread disparities across populations and jurisdictions. This highlights the paramount importance of engaging a more diverse and inclusive research workforce, including the RD population.

The COVID-19 pandemic has further perpetuated and exacerbated the unmet needs and challenges experienced by the RD community, regardless of whether they were infected with COVID-19.

First, RD has been identified as a risk factor for COVID-19 related mortality. While RD patients had a similar rate of COVID-19 infection as the general population, Chung et al. reported that RD patients were associated with an adjusted 3.4 times odds of COVID-19 related hospital mortality compared to the general population in Hong Kong (95% CI 1.24–9.41; *p* = 0.017) (39). Similar findings were observed in a retrospective cohort study in Genomics England 100k Genomes participants, in which RD patients were found to have a 3.5 times odds of COVID-19-related deaths compared to the unaffected relatives (95% CI 1.21–12.2), although the effect was insignificant after adjusting for age and number of comorbidities (OR 1.94; 95% CI 0.65–5.80) (40). COVID-19-related mortality was not confined to one specific group of RD patients, as suggested by both studies. Results from these studies suggested that RD as a group is a pre-existing comorbidity that is associated with COVID-19-related mortality, and should be considered in healthcare prioritization (39, 40).

In addition to RD patients who were infected with COVID-19, patients without infection had also experienced enormous and multifaceted challenges during the pandemic. Interruptions of care, particularly delays and cessation of diagnostic workups, therapies, rehabilitation, surgeries, and medications, pose substantial impact on the health and social wellbeing of the RD patients. Genetic laboratories and hospitals were required to provide urgent services only, to focus manpower and resources on combating COVID-19. In the United Kingdom, referrals to Clinical Genetics Service fell over 50% during April to June 2020 as compared to the same period in 2019 (41). Request for genetic testing such as microarrays, which is often the first line genetic diagnostic test for patients with suspected undiagnosed genetic disease, was markedly reduced (41). There was also substantial decrease in the number of other diagnostic tests performed, including echocardiograms, radiological investigation, and gastroscopies (41). The pandemic has disproportionately exacerbated the problem of diagnostic delay for RDs, affecting all points on the path to diagnosis, from initial engagement with health services, referral for investigation or specialist assessment, to the availability of definitive testing and registering with patient advocacy groups for support. In addition to the significant drop in RD diagnosis, health service utilization was also substantially affected. In Hong Kong, over 70% of the RD patients had reduce health service utilization during the pandemic (42). Importantly, health status was affected in 46% of the patients due to reduced service provision. Psychological health and rehabilitation were affected in 79% and 78% of the patients respectively, especially among patients who are severely or totally dependent according to the Barthel Index for Activities of Daily Living (42). Moreover, patients' social life, daily living, and financial status were also severely impacted by the COVID-19 pandemic, affecting 92%, 89%, and 81%, respectively (42). Almost 60% of the patients reported increased expenditure during the pandemic, while 56% of the patients experienced reduction in household income, indicating the magnified financial burden on the RD population (42). Similar patterns were also identified in other cohorts in the West, all highlighting the significant repercussion of the pandemic on regular healthcare service, physical and psychological health, and financial status of the RD population (43–47). The COVID-19 pandemic has inspired and accelerated the adoption of telemedicine and telehealth in some parts of the world (43, 48). Future implementation of telemedicine into the healthcare systems may serve as a sustainable healthcare delivery model beyond the COVID-19 pandemic.

For carers of the RD patients, lifelong caring has posed substantial psychological and financial burden in the best of times, and these challenges have been further exacerbated during the COVID-19 pandemic. In a study by Fuerboeter et al. to assess the mental health and overall quality of life in parents of children with rare congenital surgical diseases in Germany, the parents, especially mothers, reported severe psychosocial impairment during the pandemic (49). Parents of the RD patients had a significantly lower quality of life than parents in the control group, potentially due to the lockdown measures imposed, daily care for the patient, work-from-home measures, and the concerns of their children being at a higher risk of infection after surgeries (49). This study highlighted the need to provide support and raise awareness for parents in addition to the RD patient *via* a family-centered approach, especially during difficult periods such as the era of COVID-19 pandemic.

Besides RD patients and carers, the pandemic has also brought unprecedented challenges to RD patient organizations internationally. A multinational cross-sectional study was conducted to evaluate the impact of the COVID-19 pandemic on 80 RD organizations across 10 jurisdictions in the Asia Pacific region, namely Australia, Hong Kong, India, Japan, mainland China, Malaysia, New Zealand, the Philippines, Singapore and Taiwan (48). The study found that almost 90% of the patient organization representatives were concerned about the pandemic's impact on their organizations. In particular, over 60% and over 40% of the participants have highlighted reduction in organization capacity and funding as their biggest challenges during the pandemic respectively (48). They have also experienced difficulties in supporting their members as physical interactions were restricted. Importantly, patient group representatives underpinned the need to move toward a digitalised era, both in organization operation and healthcare, especially amidst confinement measures. In particular, operation of RD patient organizations in Australia and New Zealand were not impacted or were less affected by the pandemic as they had greater digital capacities and have digitalised their operations prior to the pandemic (48). The pandemic has brought myriad challenges to the RD patients and organizations, yet has also created opportunities by accelerating the adaption of tele-operation and telehealth, complementing face-to-face visits and consultations.

The current COVID-19 pandemic has highlighted and exposed the long-standing and fundamental challenges and healthcare needs of the RD population. The healthcare, social care, economic, and organizational challenges experienced by the RD community indicate the importance of ensuring adequate and continuity of diagnostic and priority management strategies for RDs during pandemics and beyond.

## RDs: A global public health priority

The challenges arising from the nature of RDs have led RDs to emerge as a global public health priority. Unprecedented global integration of RD research is crucial to raise awareness, enhance understanding, accelerate diagnosis, and improve treatment for RDs. Recognizing its importance, the International Rare Diseases Research Consortium (IRDiRC) was established in 2011 to facilitate international collaboration between public and private sectors, and among stakeholders active in RDs research across government research funding bodies, companies, academia, and patient advocacy organizations around the world (50, 51). The IRDiRC have set out three 10-year goals for 2017 to 2027, with the vision to enable RD patients to achieve an accurate diagnosis, and to receive appropriate care and available therapy within 1 year of seeking medical attention (50). The three IRDiRC goals are:

- To provide all individuals with suspected RDs who have seek medical attention with a diagnosis within 1 year if the RD is reported in medical literature; and to put those who remain undiagnosed in an international coordinated diagnostic and research pipeline;
- To approve a thousand new therapies for RDs, with the majority focusing on RDs without approved options; and
- To develop new methodologies for assessing the impact of RD diagnoses and therapies.

The three IRDiRC goals mainly target the healthcare challenges of RDs, with the overarching aim being to galvanize the broad RD community to enable universal diagnosis and treatment, to ensure that the programmes and interventions can reach RD patients and families, and to pose the intended positive impact on the health and wellbeing of the RD population (50, 51).

Recognizing the importance of promoting inclusion and protecting the human rights of the RD population, EURORDIS, Rare Disease International, and the Committee on Non-Governmental Organizations (NGOs) for RDs, together called for a UN Resolution for RDs in 2019, urging the 193 UN Member States of the General Assembly to adopt the Resolution by the end of 2021. This campaign targets the RD patients and families by recognizing and addressing their needs and challenges, which aims to promote greater integration of RDs in the agenda of the UN, and advances UN's commitment in achieving the Sustainable Development Goals (SDGs) of the 2030 Agenda, with the endeavor to “leave no one behind.” The UN resolution has five key asks:

- Social inclusion and participation of RD patients and families;
- Universal and equitable access of quality healthcare without having to experience financial hardship;
- Promotion of RD strategies and actions at a national level;
- Integration of RDs into UN programmes, agencies, and priorities; and
- Routine publication of UN reports for resolution progress monitoring.

The Call for UN Resolution has promoted research and global coalition to tackle the socio-economic challenges of the RD population. The UN resolution was subsequently adopted on December 16, 2021 (52); this is an important milestone toward greater awareness and recognition for the RD community, allowing implementation of international policies to address the needs and challenges of the RD population.

## The state of evidence in addressing the challenges of the RD population

National and international stakeholders across academia, health systems, governments, funding bodies, NGOs, and patient advocacy organizations have set out research projects and programmes to tackle the challenges experienced by the RD population, contributing to achieving the three IRDiRC goals and the five UN Resolution key asks. There has been tremendous progress in RD research over the past decade, especially in RD diagnoses and gene discoveries, achieved by the advancement in genomic technologies. Positive trend in RD-related therapeutic development was also observed, with the IRDiRC's 2020 goal for 200 new therapies being achieved in early 2017, three years ahead of the agenda (50). The socio-economic burden of RDs is harder to gauge and is rather limited in literature, given the collective number of unique RDs identified and the lack of standardized methodologies to collect related data.

### Improving RD diagnoses and its implications

**Figure 2:**
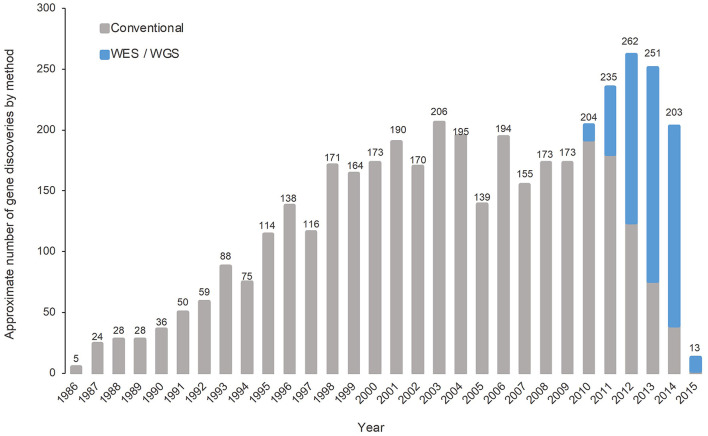
Number of gene discoveries made by WES/WGS compared with conventional diagnostic methods [adapted from Chong et al. (62)].

Traditionally, making a diagnosis is particularly challenging due to the heterogeneity and the rarity of each of the 6,000–8,000 RDs, multisystemic involvement, and pleiotropic manifestations (53–55). In the era of genomic medicine, our understanding on RDs has been transformed by the rapid expansion and translational application of next-generation sequencing (NGS) technologies in the past decade. NGS technology utilizes massively parallel sequencing methods to simultaneously and comprehensively sequence multiple genes, the entire protein-coding region of the genome (the “exome”), or the entire human genome (56). The diagnostic capacity of whole-exome sequencing (WES) and whole-genome sequencing (WGS), both NGS approaches, were shown to be effective over conventional diagnostic approaches across multiple studies in different populations. In a meta-analysis including 37 studies and comprising 20,068 children, the pooled diagnostic rates among WES and WGS were found to be 0.36 (95% CI 0.33–0.40, *I*^2^ = 83%) and 0.41 (95% CI 0.34–0.48, *I*^2^ = 44%), respectively, higher than the conventional diagnostic method of chromosomal microarray (0.10, 95% CI 0.08–0.12, *I*^2^ = 81%) (57). In critically ill patients with urgent needs, previous clinical studies have illustrated the vast amount of potential of rapid WES (rWES) and rapid WGS (rWGS) in diagnostic capacity, speed, and clinical utility in acute care (53, 58–60). The diagnostic capacity of rWES and rWGS was corroborated by findings from 18 studies comprising 1,049 patients from different countries, combined as part of a meta-analysis, with the pooled diagnostic yield being 0.43 (95% CI 0.36–0.50, *I*^2^ = 80.7%) (61). The successful application of WES and WGS in diagnosing patients with RDs in different settings has also allowed new gene discoveries over the years since its introduction in 2010 (Figure 2) (62, 63). The speed of new gene discoveries has been increasing substantially, with discoveries made by WES and WGS almost tripled the discoveries made by conventional methods since 2013 (62).

More importantly, WES and WGS offer the potential for the development of pragmatic, phenotype-driven management with genotype-differentiated personalized treatment (64). Personalized medicine, according to the National Human Genome Research Institute (NHGRI), was defined as an emerging practice of medicine that utilizes an individual's genetic profile to guide clinical decision-making in disease prevention, diagnosis, and treatment (65). WES and WGS have the potential to impact diagnosis-predicated clinical management, often referred as clinical utility, which includes but not limited to referral to specialists, surveillance for potential future complications, lifestyle changes, and indication or contraindication of investigations, procedures, surgeries, and medications (61, 66). In the meta-analysis by Clark et al. that included four WGS studies and 12 WES studies with data on clinical utility, 27% (95% CI 17–40%, *I*^2^ = 54%) and 17% (95% CI 12–24%, *I*^2^ = 76%) of children with genetic diagnoses had subsequent changes in their clinical management respectively (57). Early and rapid adoption of rWES or rWGS within a median of two to three weeks of results turnaround time could potentially impact clinical management promptly and profoundly, thus improving patient's clinical outcome and quality of life, and reducing morbidity and mortality (58–60, 67). In the intensive care setting of the National Health Service (NHS) of the United Kingdom, the use of rWGS led to changes in clinical management in 65% of the diagnosed patients (60). Chung et al. investigated the diagnostic utility of rWES and rWGS as a meta-analysis, and illustrated that genetic diagnoses could impact clinical management in up to 100% of the diagnosed patients in some cohorts (61). A rapid and timely genetic diagnosis is particularly important among critically ill patients with urgent needs, as it is potentially lifesaving.

The implication of RD diagnoses is beyond that on patients. In the era of resource and budget constraints, the evaluation of economic implications of providing WES and WGS within clinical settings has a principal role in informing efficient and effective healthcare resource allocation. Despite the high unit costs of WES and WGS, studies have demonstrated the cost-effectiveness of WES and WGS across clinical settings (68–72). On the other hand, health-economic evidence of rWES and rWGS is rather limited, with the fact that parallel comparison of rWES/rWGS and conventional diagnostic methods is more challenging due to the critical and urgent clinical setting that requires immediate clinical management decisions. Studies however illustrated the potential of rWES and rWGS to reduce healthcare costs, with costs being saved in the avoidance of unnecessary investigations, procedures, hospitalisations, and medications (58, 59, 61). In particular, Stark et al. reported a cost-saving of AU$543,178 (US$408,090) from avoidance of planned procedures and hospital days using rWES in Australia (59). In Hong Kong, Chung et al. demonstrated a reduction of 566 hospital days and a cost-saving of HK$8 million (US$1.03 million) from clinical management changes using rWES (61). In the United States, Farnaes et al. illustrated a net cost-saving of US$128,555 from reduced inpatient days using rWGS (58). Available evidence shed light on the consideration of integrating WES/WGS into clinical workflows to enable precision medicine and reduce healthcare costs.

**Figure 3:**
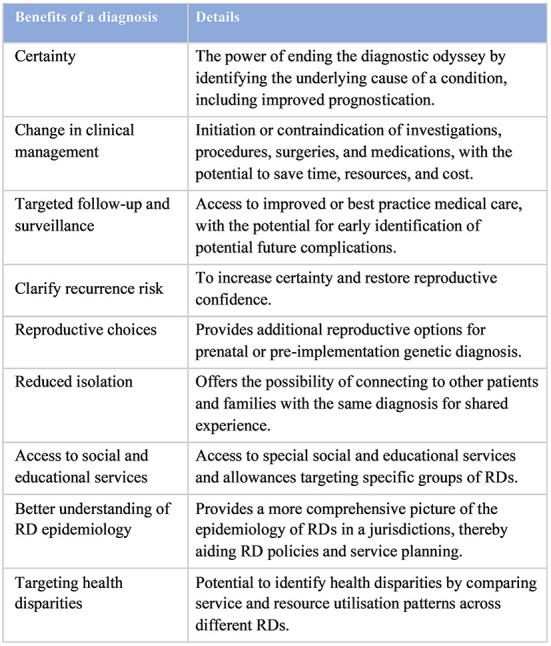
Benefits of an early genetic diagnosis [adapted from Tan et al. (73)].

The importance of an early genetic diagnosis for RD patients was demonstrated and highlighted in many of the previous studies, contributing to and reinforcing the 10-year goal of IRDiRC to provide an early definitive molecular diagnosis within 1 year of medical attention (50, 63). An accurate genetic diagnosis is the first step in managing the RD properly, allowing the identification of useful resources and treatment for the best possible clinical outcome for patients. The diagnosis-predicated changes in management not only improved clinical outcomes for patients, but could also lead to net cost-savings, stressing its multi-level significance. In addition to the immediate clinical changes and associated cost-savings from a rapid genetic diagnosis, an early genetic diagnosis has the power to aid better understanding in RD epidemiology and target health disparities, which all act as strong advocacies for the RD population (Figure 3) (73). It also contributes to existing literature and provides empirical evidence for better health- and social-care planning, such as implementation of population-wide sequencing and prevention strategies (74).

### Socio-economic costs of RDs

The societal impact of RDs has an economic dimension. In literature, majority of the economic evidence was based on individual RDs that are relatively “common,” while recent studies accounting for a wider range of RDs often quantify direct healthcare costs from a health system perspective due to the lack of standardized methodologies to collect cost-related data beyond health administrative dataset for the RD population (15, 75).

The largest study to date, estimated healthcare utilization and related costs across 1,600 RDs from a health system perspective in the United States. The study highlighted the disproportionately higher number of inpatient stays, readmissions, emergency visits, and the related costs of the RD population as compared to other common conditions (76). Similarly, the direct immediate healthcare burden of RDs was also estimated in studies conducted in Australia, Hong Kong, Shanghai, and Taiwan (77–81), suggesting the high direct healthcare costs in the RD population.

Although often neglected and rather challenging to estimate, it is also extremely important to evaluate and estimate the direct non-healthcare and indirect economic consequences for healthcare and related planning, especially in a chronic disease population. With RDs often being medically devastating and life-threatening, unpaid informal carers, usually a family member or a friend of the patient, play an extremely important role in supporting and assisting the patient's daily healthcare and social needs. This harbors a unique set of challenges and burden in RD carers, which encompasses coordination of care as well as helping with daily activities, both of which have spill over effects onto the carers' own personal lives, especially work responsibilities. The nature of RDs thus potentially inhibits patient's and carer's participation and integration into society, resulting in significant productivity loss, posing financial constraints for the RD family in addition to the substantial medical costs that often requires cost-sharing by the patient (82). On the other hand, many of these carers, usually both parents of the patients with RDs, have to sustain family's financial income by staying in the workforce. Therefore, paid carers, such as live-in domestic helpers, are commonly hired as an alternative to provide formal care support. This is particularly prevalent in Asia, such as the case in Hong Kong. In fact, previous evidence has demonstrated that direct non-healthcare and indirect costs of RDs (including paid and unpaid carers) are higher than direct healthcare costs of RDs, reflecting the importance to consider the broader socio-economic consequences of RDs in health- and social-care policies (16).

As highlighted by a meta-analysis published in 2021 that identified 19 studies in literature, economic evidence from a wider societal perspective has been very limited, with majority of the evidence focusing on individual RDs (75). Almost all of the identified studies were conducted in European populations, with many of them collected as part of the “Social Economic Burden and Health-Related Quality of Life in Patients with Rare Diseases in Europe” (BURQOL-RD) project series, which estimated the costs of 10 relatively “common” RDs across eight jurisdictions in Europe (15, 83–92). The results undoubtedly aided understanding on the patterns of resource use and areas that require prioritization, supporting appropriate healthcare planning for these 10 RDs. Nevertheless, the 10 selected RDs may be insufficient to encompass the heterogeneity and differential impacts of the 6,000–8,000 known RDs. It is important to note that the economic impact of RDs that are relatively “rare” was never reported in literature, due to the challenges in patient recruitment. Recently, the EverydayLife Foundation has published a report that estimated the costs of 379 RDs in the United States from a societal perspective, which was found to be US$62,141 per patient per year (16). In 2019, the national cost of RDs in the United States totalled US$966 billion (non-healthcare and indirect costs accounting for 56.7%), significantly higher than the costs estimated for some of the most expensive chronic illnesses, including cancer, diabetes, and heart disease as indicated by the Centers for Disease Control and Prevention (CDC) (16). Although only 379 of the 6,000–8,000 RDs were included for estimation, to the best of knowledge, this represents the only and the most comprehensive study to evaluate the socio-economic burden of RDs as a collective group.

In addition to the high societal costs of RDs, it was anticipated that the disproportionately high service and resource needs, and the RD-related productivity loss might pose significant financial burden on the RD families, putting them at a higher risk of experiencing financial hardship. Only two studies have attempted to evaluate the proportion of financial hardship brought about by extremely high OOP health expenditure in the RD population to date, one being in China where the authors have estimated the rate of catastrophic health expenditure (CHE) across seven RD groups (93), and another study being in Turkey where the authors estimated the CHE incidence mainly in patients with metabolic and neuromuscular diseases (94). These two studies have reported very different rates of CHE at different thresholds (0.0015–0.1670% vs. 47.35%), reflecting the differences in healthcare and social care contexts, and the availability and accessibility of resources across jurisdictions.

### Health-related quality of life of the RD population

The impact of RDs can also be determined by quantifying patient's health-related quality of life (HRQoL). HRQoL is defined as “an individual's perception of his/her living quality, encompassing physical, mental, and social wellbeing” (95). Most RDs are typically chronic, progressive, degenerative, and life-threatening, with effective drugs being costly and scarce. Social exclusion and discrimination based on RD health conditions further depletes available resources for coping with RDs (27). It is therefore crucial to identify and understand the impact of disease and social related difficulties on the quality of life of RD patients. Previous studies have attempted to investigate the HRQoL of the RD population in more than one RD group, and have highlighted the significantly lowered HRQoL as compared to the general population (15, 28, 95, 96). In particular, the meta-analysis by Ng et al. included four studies comprising 2,079 RD patients and demonstrated a pooled utility score of 0.57 (95% CI 0.48–0.66), consistently lower than that of the general public (95). Importantly, Ng et al. has also demonstrated the “spill over effects” on carers' HRQoL in Hong Kong. Lifelong caring, high dependency of patient, and economic strain are all factors that contribute to the decreased wellbeing of patient family members and carers. In Hong Kong, both RD patients (mean utility score of 0.53) and their carers (mean utility score of 0.78) reported lower utility scores than the general population (mean utility score of 0.92) (95). More strikingly, they reported utility scores even lower than that of patients with other chronic illnesses, including patients with heart disease (0.88), hypertension (0.88), diabetes (0.87), and cancer (0.87), reflecting the disproportionate impact of RDs on healthcare and social wellbeing (95, 97, 98).

## The role of Genome Projects in advancing genomic medicine

The importance of generating greater representation and diversity across genomic datasets is becoming more widely recognized. Initially, genetic research and genomic databases were biased toward data from Caucasians, particularly of European ancestry. In 2009, 96% of genome-wide association studies were of European descent (99). Groups of other ancestries were very poorly represented. The lack of ethnic diversity in genomics was limiting the usefulness of genomic technologies and widening inequalities across different populations. To address this, contribution of genomic data of other ethnicities has increased over the past few years, increasing from 4% in 2009 to 19% in 2016 (99).

Besides research, governments have also started to launch Genome Projects in their respective jurisdictions to apply WGS to the study of RDs, and to a lesser extent, cancers and common disorders, at a much bigger population size, or even at a nationwide level, to integrate genomic medicine into mainstream healthcare and to improve global genomic diversity and equity (100).

**Figure 4:**
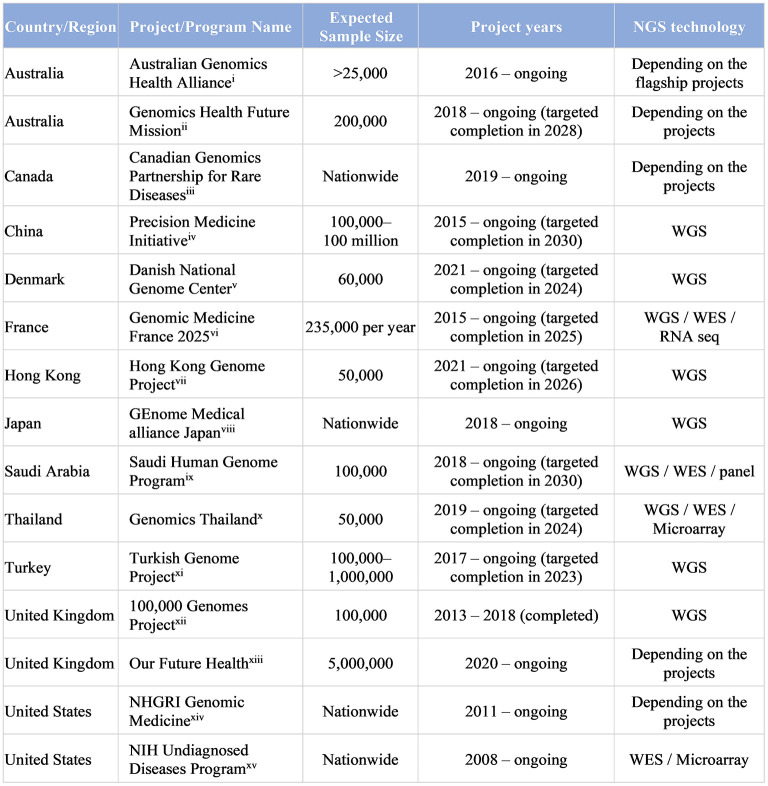
Large-scale Genome Projects targeting RDs and undiagnosed diseases (>20,000 subject genomes) (i) (104, 105); (ii) (106); (iii) (107); (iv) (108, 109); (v) (110); (vi) (111); (vii) (112); (viii) (113, 114); (ix) (115); (x) (116, 117); (xi) (118, 119); (xii)(101, 120); (xiii) (121, 122); (xiv) (123); (xv) (124). [adapted from Chung et al. (100) and Chu et al. (103)].

The government of the United Kingdom has launched the 100,000 Genomes Project in 2013, and it has been a huge success in providing grounds for the NHS Genomic Medicine Service to be the first national health care system to offer WGS as part of routine clinical care for patients with undiagnosed RDs and cancers (101, 102). This has inspired governments worldwide, even in middle-income countries, to launch Genome Projects in their respective jurisdictions, aiming to enhance clinical application of genomic medicine for personalized medicine (Figure 4) (100, 103). In the upcoming years, results from Genome Projects worldwide would potentially enhance our capability to better diagnose and manage RDs, and would provide empirical evidence for implementation of WES/WGS in health systems. More importantly, genomic data across populations, especially those beyond Europe and North America, will together contribute to improving equity of access and return in global genomics.

### Case study: The Hong Kong Genome Project

As discussed above, genomic data of non-European ancestries has been increasing over the years. Genome Projects in Asia for example, are playing a major role in contributing genomic data of Asian ancestry to improve global genomic diversity. In Asia, Hong Kong has a relatively homogeneous Chinese population. The case study of the Hong Kong Genome Project (HKGP) was selected to illustrate the contribution of Chinese genomic data.

In the 7.5 million population in Hong Kong with 94% of the population being Chinese (ethnically speaking, Han Chinese), one in 67 individuals is living with one or more RDs, with 35% being pediatric patients (78, 125). As of 2018, over 470 RDs have been identified in Hong Kong, affecting approximately 1.5% of the population (78). In order to enhance clinical application of WGS to benefit patients and families, particularly the RD population, and to strive for excellence and adherence to international standards, the Hong Kong Genome Institute (HKGI) was established in May 2020 by the former Food and Health Bureau (currently the Health Bureau), Hong Kong Special Administrative Region, to implement the HKGP, with the vision being “to avail genomic medicine to all for better health and wellbeing” (126).

The HKGP, which is implemented in two phases, the pilot phase and the main phase, is the first large-scale genome sequencing project in Hong Kong. It is set to conduct WGS for 20,000 cases with the aim to enhance clinical application of genomic medicine to benefit patients and their families with more precise diagnoses and personalized treatment (126). The pilot phase of the HKGP was launched in July 2021, focusing on undiagnosed diseases and hereditary cancers. Lessons learnt during the pilot phase of HKGP would guide the directions of the Project's main phase, which is set out to be rolled out in July 2022, expanding eligibility to cover other hereditary diseases and research cohorts related to “genomics and precision health.”

With WGS being offered as part of the HKGP, it could potentially lead to diagnosis-predicated precision medicine for patients and families, thereby improving patient outcomes whilst minimizing healthcare expenditure and related financial hardships, achieving diagnostic, clinical, and economic utility. In addition to the clinical benefits, the HKGP also aims to advance research, establish infrastructure and protocols, nurture talents, enhance public genomic literacy and engagement, and drive health- and social- care policy measures to pioneer the development of genomic medicine in Hong Kong (126). The potentials and prospects that have emerged in the launching of the HKGP pilot phase were highlighted by Chu et al., providing insights to prepare for the launching of the main phase (103). With HKGP being the first and the largest local clinical genomic database, it creates novel research opportunities for studying various diseases, including RDs, contributing to improving genomic equity in healthcare. With a relatively homogenous population, genomic data generated from the HKGP would contribute to global genomic diversity in the foreseeable future.

## Future directions of RDs

With the three 10-year goals laid out by IRDiRC and the adoption of the UN Resolution in December 2021 to stress the importance of including RD population in the UN 2030 Agenda, great strides have been made in RD research over the past decade, presenting a major shift in the global policy landscape. In particular, previous studies have highlighted the importance of an early diagnosis and the significant consequences of RDs on the living quality and socio-economic burden of patients. It should be recognized that the health, social, and economic implications of RDs are inherently the results of insufficient social support, limited medical expertise, and the lack of public awareness on RDs. As RD emerges as a global public health priority, RD policies and strategies in the sectors of healthcare, social care, insurance, education, and many more, are required to foster a more equitable and inclusive community for the RD population. Progress in RD research and analysis will likely improve all disease understanding in the future. Here, we recommend future action plans for RDs through a patient-centered and multidisciplinary approach, focusing on the implementation of education and training programmes, elimination of discrimination and stigmatization, and global coalition among multi-disciplinary stakeholders.

Firstly, providing education and training to clinicians at the primary, secondary, and tertiary care level is the first step to an accurate genetic diagnosis. Despite the technological advancement and increased data sharing, many RD patients still experience extensive diagnostic odysseys, and some remain undiagnosed. One of the major barriers to obtaining a diagnosis is the lack of knowledge and insufficient training on RDs. According to the National Organization for Rare Disorders (NORD) survey 2019, almost half of the patients and carers identified limited medical specialization to be a major barrier to delays in RD diagnosis (127). Previous studies have shown that many primary care physicians profess low confidence in their skillsets in managing patients with genetic-related issues and in using genetic information to make clinical decisions (128–131). Primary care physicians have identified lack of knowledge and training opportunities to be the major barriers to genomic medicine in primary care (128, 131, 132). Emphasis on further education and training in genomic medicine among medical specialists should be prioritized in order to improve RD diagnosis and management. Continuous technological and technical advancement in genomics is also required to diagnose patients and to transform sequencing information into diagnostic knowledge, such as the application of bioinformatics, analytic algorithm, functional analysis, health informatics, data linkage capability, data sharing, etc. (74, 133). Global network involving full participation by clinicians, researchers, and patients and carers should be formed to tackle the undiagnosed cases (74). International efforts have been made over the years to investigate and diagnose patients who had long sought one without success, such as the initiation of the Undiagnosed Disease Program by the National Institutes of Health (NIH) and the Undiagnosed Diseases Network International (134, 135). The establishment of these programmes and networks have supported global improvements in diagnosis of RDs *via* core principles and implementation methods (74).

Secondly, in addressing the significant socio-economic burden and the lowered HRQoL of the RD population, the government and healthcare system should work together to provide affordable and accessible resources, thereby improving the HRQoL of patients and carers. Previous cost-of-illness studies highlighted the unique and complex challenges the RD population face, providing strong evidence that management of these challenges should be treated differently to other common disease (15, 16, 77–79, 83–92). With RD patients requiring services and care across multi-disciplines, patients often experience frustration in service fragmentation. The implementation of “one-stop” clinics may improve coordination of care through providing various services at a single location, tackling multiple and complex problems simultaneously (29). In France and the United Kingdom, the integration of “one-stop” clinics was shown to improve coordination between services, providing timely and informed care to the RD population (136). Overall, the utilization of such “one-stop” clinics have yielded better patient outcomes and are more cost-effective and thus, are a possible solution to the high socio-economic burden and the lowered HRQoL of RDs (137). In addition, implementation of reimbursement regulations would improve affordability and accessibility of treatments, potentially reducing the risk of financial hardship of the RD population (138). Other action plans in Australia and New Zealand have also been put forward to support the RD population (139). These aim to reduce the healthcare, social care, and economic burden through empowering, improving diagnosis and intervention, coordinating care and increasing research for RDs.

Thirdly, the government plays a pivotal role in raising awareness and in mitigating discrimination and stigmatization of the RD population. Efforts have been made in different parts of the world to implement Genome Projects to integrate genomic medicine in mainstream healthcare. In order to enhance understanding and mitigate genetic discrimination, unprecedented global coalition is of paramount importance to improve inclusivity of the RD population. Previous studies have demonstrated participants' concerns on genetic discrimination in the context of employment and insurance (140–142). In particular, undergraduates in Hong Kong were found to be pessimistic toward unfavorable genetic testing results, with almost 60% of the respondents claiming that they would feel “inadequate or different,” 56% would feel helpless, and nearly 60% perceived that they would be disadvantaged in job seeking in case of unfavorable genetic testing results (140). It is of utmost importance to eliminate the root causes of stigmatization and discrimination of the RD population in order to improve social inclusion and reduce opportunities and productivity loss. This can be done through the implementation of anti-discrimination policies such as Genetic Information Nondiscrimination Act (GINA) in the United States to aid assimilation of the RD population into society (143, 144). Legislations in Japan and Taiwan have also incorporated social care services into their RD framework, thereby facilitating the inclusion and integration of the RD population into the society in addition to providing quality healthcare (145). Additional education and awareness for the public on the RDs should also be implemented to increase acceptance and reduce stigma. Both these strategies in conjunction will work toward improving social integration of the RD population, thereby improving their HRQoL and reducing socio-economic burden.

Fourthly, a more widespread utilization of telehealth or telemedicine constitutes a sustainable and alternate model amidst the COVID-19 pandemic and beyond. Telemedicine has the potential to revolutionize patient access to clinical specialists around the world without geographical boundaries. The COVID-19 pandemic has accelerated the digitalisation of healthcare across the world and have inspired healthcare professional licensing agencies to address this in various nations and states. This improves access for all patients in both urban and rural areas, and in both developed and developing countries regardless of their economic status. Adoption of telemedicine into routine clinical care would require innovative approaches to increase capacity and the strengthening of health systems. In the long run, greater reliance on telemedicine is undeniably the way forward, which constitutes a sustainable healthcare delivery model in times of and beyond pandemics.

Finally, RD patient organizations have the power to drive forward the adoption of necessary policies and help to coordinate care (146). Governments should strive to strengthen the public's awareness on the needs of RD populations through in-depth conversations and focus group meetings with patient representatives. On one hand, RD patient groups have important roles in advocating for patients' rights and research opportunity. On the other hand, patient groups are the pillar of psychologic support for patients and their families. The recently adopted ground-breaking UN Resolution led by Rare Disease International, EURORDIS, and the Committee on NGOs for RDs serves as a strong example. It represents a major shift in the global policy landscape, by promoting greater integration and prioritization of the RD population in the UN agenda. Through this global campaign, the needs of the RD community are brought to light, allowing for the development of necessary strategies and plans to provide affordable and accessible care. Acting as the voice of the RD population, RD patient organizations can empower patients and carers alike while raising awareness to educate the community.

Taken collectively, there is a scientific, social, ethical, and political imperative to promote greater integration and inclusiveness of RDs in research and policies, contributing to the goal of the UN Resolution, to “leave no one behind.”

## Author contributions

CC and BC contributed to the conception of the review. CC performed the literature review and drafted the manuscript. Hong Kong Genome Project, AC, and BC critically reviewed and revised the manuscript for important intellectual content. AC and BC oversaw and supervised the review. All authors contributed to the overall interpretation, reviewed, and approved the final draft for submission.

## Conflict of interest

The authors declare that the research was conducted in the absence of any commercial or financial relationships that could be construed as a potential conflict of interest.

## Publisher's note

All claims expressed in this article are solely those of the authors and do not necessarily represent those of their affiliated organizations, or those of the publisher, the editors and the reviewers. Any product that may be evaluated in this article, or claim that may be made by its manufacturer, is not guaranteed or endorsed by the publisher.

## Abbreviations

NHGRI: National Human Genome Research Institute
NIH: National Institutes of Health
RD: rare disease
RNA Seq: RNA sequencing
WES: whole-exome sequencing
WGS: whole-genome sequencing.
